# Supplementary material for: Early temperament and physical health in school-age children: Applying a short temperament measure in a population-based cohort
Source: PLoS One. 2023 May 22;18(5):e0285710. doi: 10.1371/journal.pone.0285710 (PMC10202300; doi:10.1371/journal.pone.0285710)
Supplement: S1 Table — (DOCX) [file pone.0285710.s001.docx]

**S1 Table. The dimensions and corresponding questions of the TBCS short temperament measure**

| **Dimension** | **Question and rating scale** |
| --- | --- |
| Activity | Your child is energetic and physically active, OR is quieter and does not enjoy games involving physical movement.  (1=low activity level; 7=high activity level) |
| Rhythmicity | Your child eats, sleeps, and wakes up, etc. at regular times, OR you find yourself unsure of what times your child will eat, sleep, and wake up, etc. (1=low regularity level; 7=high regularity level) |
| Approach | Your child approaches and enjoys new people, situations or foods, OR is shy and hesitant in accepting these interactions or situations.  (1=highly shy; 7=highly outgoing) |
| Adaptability: | Your child adapts quickly to new environments or things, OR requires some time to adapt to new environments or things.  (1=low adaptability level; 7=high adaptability level) |
| Mood | Your child mainly smiles and is in pleasant mood, OR has an unpleasant expression and seems unhappy.  (1= unpleasant and negative mood; 7= pleasant and positive mood) |
| Persistence | Your child only moves on from a task once having finished it entirely, is persisting even encountering difficulties, OR gives up or interrupt tasks before completing them.  (1=low persistence level; 7=high persistence level) |
| Attention | Your child can concentrate on doing a task, OR is easily disturbed by external stimuli then distracted.  (1=high distractibility level; 7=high attention level) |
| Threshold of responsiveness | Your child is easily affected by sensory stimuli such as light, noises, other people's emotions or minor changes in appearance, OR tends to be unaffected or unresponsive.  (1=low sensitivity; 7=high sensitivity) |
| Intensity | Your child expresses strong reactions, such as laughing or crying, OR shows relatively mild emotional reactions.  (1=low emotional reactivity; 7=high emotional reactivity) |
