# Supplementary material for: Early temperament and physical health in school-age children: Applying a short temperament measure in a population-based cohort
Source: PLoS One. 2023 May 22;18(5):e0285710. doi: 10.1371/journal.pone.0285710 (PMC10202300; doi:10.1371/journal.pone.0285710)
Supplement: S2 Table — (DOCX) [file pone.0285710.s002.docx]

**S2 Table. Correlation coefficients between the items and temperament trait scores of the TBCS short measure (N=18,944)**

|  | 1 | 2 | 3 | 4 | 5 | 6 | 7 | 8 | 9 | 10 | 11 |
| --- | --- | --- | --- | --- | --- | --- | --- | --- | --- | --- | --- |
| 1. Activity | 1.00 | - | - | - | - | - | - | - | - | - | - |
| 2. Rhythmicity | 0.07^***^ | 1.00 | - | - | - | - | - | - | - | - |  |
| 3. Approach | 0.33^***^ | 0.12^***^ | 1.00 | - | - | - | - | - | - | - | - |
| 4. Adaptability | 0.30^***^ | 0.16^***^ | 0.62^***^ | 1.00 | - | - | - | - | - | - | - |
| 5. Mood | 0.25^***^ | 0.22^***^ | 0.35^***^ | 0.39^***^ | 1.00 | - | - | - | - | - | - |
| 6. Attention | -0.05^***^ | 0.25^***^ | 0.07^***^ | 0.14^***^ | 0.18^***^ | 1.00 | - | - | - | - | - |
| 7. Persistence | 0.06^***^ | 0.21^***^ | 0.11^***^ | 0.16^***^ | 0.14^***^ | 0.46^***^ | 1.00 | - | - | - | - |
| 8. Sensitivity | 0.10^***^ | 0.23^***^ | 0.17^***^ | 0.22^***^ | 0.20^***^ | 0.30^***^ | 0.30^***^ | 1.00 | - | - | - |
| 9. Intensity | 0.18^***^ | 0.02^*^ | 0.09^***^ | 0.04^***^ | 0.02^**^ | -0.02^**^ | 0.05^***^ | 0.13^***^ | 1.00 | - | - |
| 10.. Surgency trait | 0.62^***^ | 0.20^***^ | 0.83^***^ | 0.81^***^ | 0.65^***^ | 0.12^***^ | 0.16^***^ | 0.23^***^ | 0.11^***^ | 1.00 | - |
| 11. Regulation trait | 0.04^***^ | 0.30^***^ | 0.15^***^ | 0.23^***^ | 0.23^***^ | 0.80^***^ | 0.79^***^ | 0.68^***^ | 0.07^***^ | 0.22^***^ | 1.00 |

*<.05; **<.01; ***<.001.
