# Supplementary material for: Early temperament and physical health in school-age children: Applying a short temperament measure in a population-based cohort
Source: PLoS One. 2023 May 22;18(5):e0285710. doi: 10.1371/journal.pone.0285710 (PMC10202300; doi:10.1371/journal.pone.0285710)
Supplement: Attachment — Submitted filename: Review.docx [file pone.0285710.s004.docx]

**Review**

65. You spelt out a disorder associated with externalizing behaviors – ADHD, what would be a disorder associated with internalizing behaviors “ Depression, Adjustment etc”

152. It would be clearer/easier for a reader if you included years during which follow-ups were done.

159. This study **“was”** delete **“has been”**

252. Remove “ay”

188 -189. How did you take care of a response from a caregiver in lower social strata Vis one from higher socioeconomic strata, regarding possible differences in evaluating what constitutes a good or poor health?

Overall

A very detailed study, topic in sync with the objectives, analysis and discussions.

Take care of typos e.g.

50. could delete “such as” or insert “measurements” to read as ** Measurements such as**
