# Supplementary material for: Early temperament and physical health in school-age children: Applying a short temperament measure in a population-based cohort
Source: PLoS One. 2023 May 22;18(5):e0285710. doi: 10.1371/journal.pone.0285710 (PMC10202300; doi:10.1371/journal.pone.0285710)
Supplement: Attachment — Submitted filename: Respose to Reviewers_r2.docx [file pone.0285710.s005.docx]

We would like to thank the reviewers for raising a number of critical points. All these have helped us to ensure that the main study objective holds while the reason of validating a short temperament measure is made clear. Point-by-point responses are provided below, and the changes in the revised manuscript are highlighted in blue.

Reviewer #1: The authors have taken most of my comments into account. However, some of their responses have raised new comments that must be addressed before the manuscript is suitable for publication.

1. The modification made in the introduction need to be revised. Indeed, it is not because you used large scale longitudinal data that you need to validate the questionnaire that was implemented. You chose in the design of TBCS to implement a new questionnaire. You need to validate it before analyzing it.

**Response**: Thank you. We have made it clear that the need and purpose of validating the short temperament measure of TBCS is to apply it for further investigation of our research question. (lines 90-93)

2. I understand the usefulness of using a short scale in large cohort however, the explanation provided does not really show any plus in using this scale compared to other already existing short ones.

**Response**: Thank you and we agreed with you on this comment. The sentences in the Introduction that we attempted to explain the usefulness of developing a new short scale of temperament have been removed.

3. The added sentence on the effect size in the method section lines 138-140“It is anticipatory given that single-item measures are unable to fully capture complex constructs and temperament is one example that generally relies on multiple items to reflect child’s behaviours and reactivity in various contexts” is confusing since temperament is the main topic of this validation study and you write lines 130-131 “ … providing evidence for the applicability of the nine-item temperament measure”. This should be rephrased.

**Response**: Thank you. The paragraph has been rephrased as “It is anticipatory given that temperament generally relies on multiple items to reflect child’s behaviours and reactivity in various contexts and single-item measures are unable to fully capture such a complex construct.” to avoid confusion. (lines 141-143)

4. I understand the interest in using higher order temperament factors. However, the entire introduction section focused toward this new 9-item scale and nothing prepares for the higher order factors that are finally the main results presented. Thus, as written, it seems like because nothing came out at the first analysis using the nine items you finally group them to hopefully find associations with health outcomes.

**Response**: We chose to derive higher-order factors in our analysis. The decision was made by comparing the advantages of using higher-order versus lower-order temperament traits and considering the plausibility of how temperament is related to physical health outcomes of interest. Thank you for reminding us of missing this piece of information which we have added in the Introduction. (lines 94-102)

5. Why not the same adjustment factors in both models? Why the maternal age was not included? The observed relations can be affected by shared measurement errors but not only. There are some confounders that were not considered, this should be acknowledged and discussed in the limitation section.

**Response:** Thank you. The ecosystem model of child health provided us with an overall framework for establishing the regression models. Child injury is an important health issue which specific etiological models and preventive measures are usually built for. Thus, we considered it more appropriate to include a slightly different set of covariates in the model for injury occurrence. How the covariates were selected has been modified and described in more detail. (lines 194-199). The question about maternal age is also well noted, given that mother’s age may have an influence on their perception of child’s health, childrearing attitudes and practices. In the newly calculated models, we included maternal age as a covariate and the main results remained unchanged. (Table 5.) Some potential confounders omitted from the analysis have been acknowledged in the Discussion. (lines 318-322)

6. Still many typos, e.g., temperature, table 5 age 5 and 5.5…

**Response**: Thank you. We have checked the text and tables carefully throughout the manuscript to avoid typos.

Reviewer #2: None

Reviewer #3:

1. You spelt out a disorder associated with externalizing behaviors – ADHD, what would be a disorder associated with internalizing behaviors “ Depression, Adjustment etc”

**Response**: Thank you. According to the references cited (citation [3,4]), we added in the text “anxiety or depression” which are psychopathological problems associated with internalizing behaviours and most addressed in the temperament literature. (lines 65-66)

2. It would be clearer/easier for a reader if you included years during which follow-ups were done.

**Response**: Thank you. We have specified the period of data collection for the baseline and each follow-up surveys. (lines 152-153)

3. This study “was” delete “has been”

**Response**: Thank you. The grammatical error was corrected. (line 159)

4. Remove “ay”

**Response**: “ay” is a typo for “at” and has been corrected. (line 252)

5. How did you take care of a response from a caregiver in lower social strata Vis one from higher socioeconomic strata, regarding possible differences in evaluating what constitutes a good or poor health? (line188-189)

**Response**: Thank you. Indeed, there is evidence that individuals from different socioeconomic groups (e.g. education or income levels) may rate their health differently due to differential reporting standards or the content of health evaluated. Similar report patterns may also exist when caregivers evaluate child’s health on a subjective rating scale. By adjusting for caregiver's level of education in the regression models, we assumed this problem could be somewhat mitigated.

6. Take care of typos e.g. could delete “such as” or insert “measurements” to read as ** Measurements such as** (line 50)

**Response**: Thank you. We have checked the text and tables carefully throughout the manuscript to avoid typos.
